# Physiological Studies and Ultrastructure of *Vigna sinensis* L. and *Helianthus annuus* L. under Varying Levels of Nitrogen Supply

**DOI:** 10.3390/plants11141884

**Published:** 2022-07-20

**Authors:** Khadiga Alharbi, Samia A. Haroun, Amany M. Kazamel, Mohammed A. Abbas, Safia M. Ahmaida, Muneera AlKahtani, Latifa AlHusnain, Kotb A. Attia, Khaled Abdelaal, Rasha M. E. Gamel

**Affiliations:** 1 Department of Biology, College of Science, Princess Nourah bint Abdulrahman University, P.O. Box 84428, Riyadh 11671, Saudi Arabia; mdfkahtani@pnu.edu.sa (M.A.); laalhusnain@pnu.edu.sa (L.A.); 2 Botany Department, Faculty of Science, Mansoura University, Mansoura 35516, Egypt; samiiaharon@gmail.com (S.A.H.); dr.amkazamel@yahoo.com (A.M.K.); mohammedaabbas@gmail.com (M.A.A.); dr_rashaeid@yahoo.com (R.M.E.G.); 3 Botany Department, Faculty of Science, Omar Al-Mokhtar University, Bayda 991, Libya; safiaahmaida@yahoo.com; 4 Center of Excellence in Biotechnology Research, King Saud University, P.O. Box 2455, Riyadh 11451, Saudi Arabia; kattia1.c@ksu.edu.sa; 5 Rice Biotechnology Lab, Rice Department, Field Crops Research Institute, ARC, Sakha 33717, Egypt; 6 Excellence Center (EPCRS), Plant Pathology and Biotechnology Laboratory, Faculty of Agriculture, Kafrelsheikh University, Kafr Elsheikh 33516, Egypt

**Keywords:** nitrogen fertilizers, total soluble-N, sunflower, cowpea, ultrastructure, transmission electron microscopy

## Abstract

This experiment was conducted to investigate the effects of different nitrogen fertilizers (potassium nitrate and/or urea) on shoot parameters, relative growth rate, net assimilation rate, and nitrogen fractions, as well as to conduct transmission electron microscopy, of *Vigna sinensis* L. (cowpea) and *Helianthus annuus* L. (sunflower) leaves. A general improvement was recorded in the shoot parameters of the two plants, except for a decrease in the net assimilation rate by treatment of the two plants with 100% potassium nitrate plus 100% urea. The total nitrogen, insoluble protein, and total soluble nitrogen generally decreased in cowpea shoots from the treatments but increased in case of cowpea roots and sunflower shoots and roots. The examination of the ultrastructure changes in cowpea leaves confirmed the presence of two starch granules (in response to 100% potassium nitrate, 100% potassium nitrate plus 100% urea, and the control) and three granules (in response to 50% potassium nitrate plus 50% urea) and the disappearance of the starch granules (in response to 100% urea). Despite the starch granules not being detected in the leaves of the untreated sunflower, the treated plant showed the appearance of the highest number after treatment with 50% potassium nitrate plus 50% urea (2) and the most cell size with the 100% potassium nitrate treatment. Generally, our findings demonstrated that fertilization with 50% potassium nitrate plus 50% urea has the best influence on the growth parameters and nitrogen content in the two plants, but the magnitude of response was more pronounced in case of cowpea plants.

## 1. Introduction

*Vigna sinensis* L. (cowpea) is one of the most ancient food sources and has likely been used as a crop plant since Neolithic times [1]. It is an important commodity in West Africa. It belongs to *Fabaceae* family, which contain many important crops such as the faba bean [2,3], soybean [4,5], and pea [6,7,8]. The cowpea is an important source of protein, vitamins, and income for humans [9]. As in many other legumes, the seeds are the most economically valuable part of the cowpea and are well-known for their nutritional and medicinal properties. Known to be an excellent source of protein, the cowpea is also rich in important vitamins, minerals, and soluble and insoluble dietary fiber. All parts of cowpea plants are used for food or fodder, and plant residues are used as fodder for farm animals [10]. The cowpea is an important African food legume suitable for dry regions [11]. 

*Helianthus annuus* L. (sunflower) belongs to the family *Asteraceae*. Eastern North America is reported as its origin [12]. It is an important oil crop and its oils exhibit antioxidant characteristics [13]. The seed yield of a sunflower has improved over the years because of the increasing demand for its healthful oil, which contains minerals like magnesium, protein, phytonutrients, and phenolic antioxidants [14,15]. Fertilization is the main tool of agricultural engineering, and it has a strong effect on the productivity, nutritional quality management, and regulation of harvest processes [16]. Nitrogen is one of the most significant elements of soil fertility, and the application of the chemical N has led to increased nitrogen levels in soil and, consequently, increased crop yields in many plants [17]. 

Nitrogen is an essential macro-element and the fourth most abundant plant element. It is a major constituent of several important plant substances and is considered the main fertilizer for numerous plants [18,19,20]. Nitrogen compounds comprise 40 to 50% of the dry matter of protoplasm, and it is a constituent of amino acids, the building blocks of proteins [21]. Nitrate is a polyatomic ion with the molecular formula NO_3^−^_ and a molecular mass of 62.0049 g/mol [22]. Nitrogen is utilized by plants from soil through their roots primarily in the form of nitrate (NO_3^−^_), which is the only anion used by plants in large amounts [21]. The main nitrate fertilizers are ammonium, sodium, potassium, and calcium salts. Several million kilograms of fertilizers are produced annually for this purpose [23]. Urea, or carbamide, is an organic compound (CO (NH_2_)_2_) with the greatest nitrogen amount (46% nitrogen) among all the nitrogen fertilizers, and it is called “Sugar fertilizer” [24]. A problematic issue in agriculture is the high quantity of ammonia being released, resulting in a partial loss of nitrogen from urea fertilizers [25]. Although studies on the N requirements of a number of crop plants have been conducted, insufficient reports are available and little work has been established in the role of N fertilizers on the ultrastructure (micromorphology) of leaves. Therefore, the main goal of this study was to evaluate the effects of two levels (100% and 50% of the recommended dose) of nitrogen fertilizers (potassium nitrate and urea), single or combined, on the nitrogen content of shoots, as well as on the ultrastructures, of leguminous and non-leguminous plants (*V. sinensis* and *H. annuus*), respectively. Keeping in mind that the excessive usage of chemical N fertilizers and the consequential environmental risks are very important problems in developing countries, the need for undertaking this study is justified. 

## 2. Results

### 2.1. Changes in Growth Parameters

Examination of the obtained data in this study established that a decline in some of the determined shoot growth parameters was detected in the number of nodes and leaves treated with 100% urea, 50% potassium nitrate plus 50% urea, and 100% potassium nitrate plus 100% urea and in the leaf area of plants treated with 100% potassium nitrate and 100% urea. Meanwhile, the other treatments improved the shoot growth parameters of cowpea plants, as compared to the control treatment. A general stimulation was recorded in all the determined shoot growth parameters of sunflowers compared to the control values. The relative growth rate and net assimilation rate increased with all treatments, except for the net assimilation rate in the cowpea and sunflower plants treated with 100% potassium nitrate plus 100% urea (Table 1).

### 2.2. Changes in Nitrogen Fractions

A significant decrease was detected in the total N, insoluble protein N, and total soluble N of the cowpea shoots (Table 2) that underwent the used treatments, except for the 50% potassium nitrate plus 50% urea treatment, which increased these metabolites. The used treatments significantly increased the nitrogen metabolites in cowpea roots. As compared to the control values, the total nitrogen, insoluble protein nitrogen, and total soluble nitrogen of sunflower shoots increased with the used treatments. The nitrogen metabolites in sunflower roots decreased non-significantly with 100% potassium nitrate only, whereas the other treatments increased these metabolites significantly (Table 3). 

### 2.3. Changes in Leaf Ultrastructure

**Figure 1:**
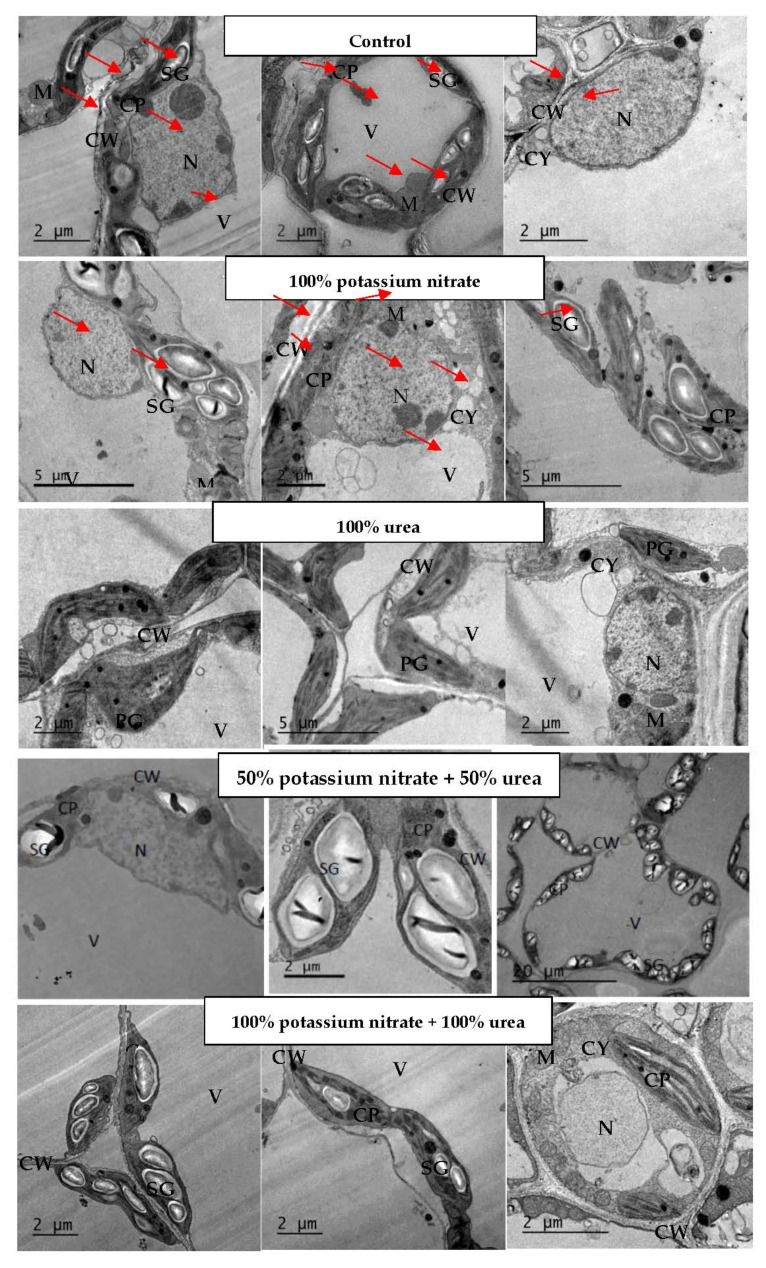
Ultrastructures of mesophyll cells of *V. sinensis* leaves at the vegetative stage (after 50 days from sowing) in response to the two levels of potassium nitrate and/or urea. V: vacuole; CW: cell wall; CP: chloroplast; N: nucleus; NU: nucleolus; CY: cytoplasm; M: mitochondria; SG: starch granules; PG: plastoglobuli.

According to the ultrastructure of the cellular and subcellular structures of *V. sinensis* leaves (Table 4 and Figure 1), treatment with 100% potassium nitrate led to a decrease in the cell size, cytoplasm, and vacuoles, and it increased the other measurements. Treatment with 100% urea decreased only the mitochondrial measurements and increased the others. The response to treatment with 50% potassium nitrate plus 50% urea had the same trend as in the case of 100% urea, except for the mitochondrial number, which increased after treatment with 50% potassium nitrate plus 50% urea. Meanwhile, treatment with 100% potassium nitrate plus 100% urea non-significantly decreased the size of cells, cytoplasm, vacuoles, and nucleus and mitochondrial items; increased cell wall thickness; and, in particular, significantly increased chloroplast measurements. Regarding starch grains, although the treatments with 100% potassium nitrate and 100% potassium nitrate plus 100% urea had the same starch granules as the control (two grains), treatment with 100% urea cleared undetected starch grains and the number of grains increased to three after treatment with 50% potassium nitrate plus 50% urea. It is of interest to notice that the total size of the starch grains increased in the detected treatments as compared to the control value.

**Figure 2:**
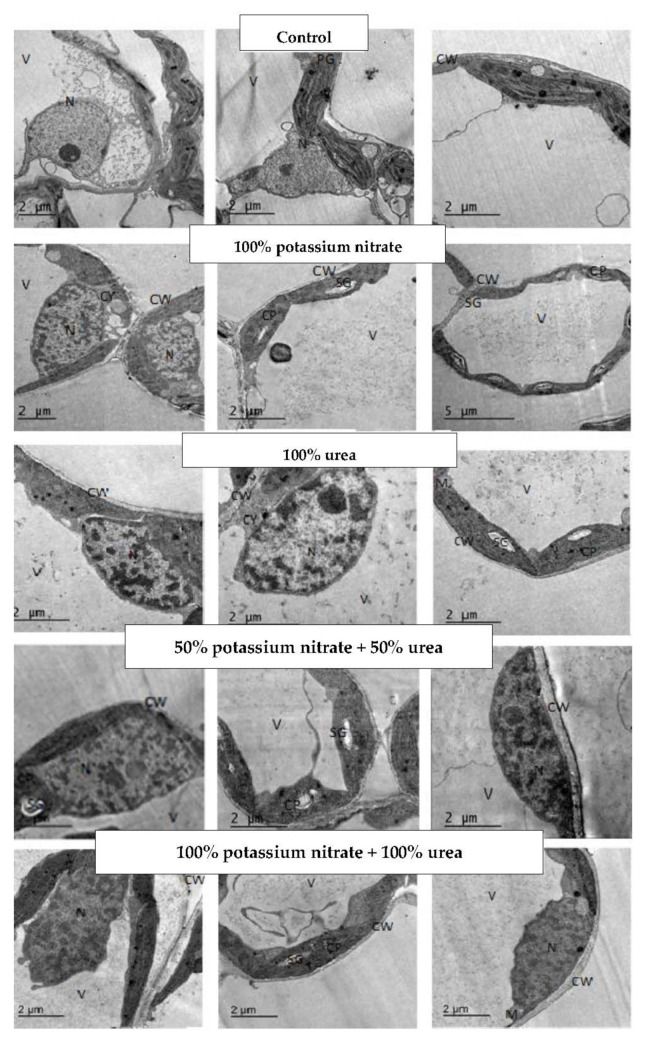
Ultrastructure of mesophyll cells of *H. annuus* leaves at the vegetative stage (after 50 days from sowing) in response to different nitrogen fertilizers (potassium nitrate and/or urea) and their combination. V: vacuole; CW: cell wall; CP: chloroplast; N: nucleus; NU: nucleolus; CY: cytoplasm; M: mitochondria; SG: starch granules; PG: plastoglobuli.

Concerning the treatment of an *H. annuus* plant with 100% potassium nitrate, a marked increase in the size of cells, mitochondria, cytoplasm, nuclei, vacuoles, and cell wall thicknesses, as well as number and size of chloroplasts, were detected, but the mitochondrial number was decreased. On the other hand, 100% urea treatment led to decreases in the studied parameters, except for cell wall thickness and vacuole and mitochondria size, which increased with this treatment. As regards the 50% potassium nitrate plus 50% urea treatment, the determined parameters either decreased (cell size, cell wall thickness, cytoplasm, vacuole and nucleus size, and chloroplast size) or increased (chloroplast number, chloroplast total size and number, and size and total size of mitochondria). Except for the decrease in chloroplast and mitochondria number after treatment with 100% potassium nitrate plus 100% urea, the other parameters increased as compared to the control values. Despite the non-detected starch grains in the leaves of the untreated sunflowers, the treated leaves showed the appearance of starch grains and had the highest number after treatment with 50% potassium nitrate plus 50% urea and the largest size after treatment with 100% potassium nitrate (Table 5 and Figure 2). 

## 3. Discussion

### 3.1. Changes in Growth Parameters

Nitrogen is a very significant element in crop production through its improvement of growth characters and productivity. The changes in the determined growth parameters in this study are in harmony with those of Caliskan et al. [26], who indicated that biological N fixation begins approximately 2 weeks after planting [27]. Therefore, adding a small dose of N at planting, called ‘‘starter N’’, is helpful for enhancing early plant growth and yield [28]. The application of a small dose of starter N is suitable for improving early growth. However, higher levels of starter N can be damaging for the biological N fixation and nodulation of plants [29,30]. In this respect, N has a vital role in improving growth characters [31]. Aminifard et al. [32] indicated that the lowest pepper plant height and number of lateral stems were detected in control plants at the vegetative and flowering stages. These results were in agreement with Bowen and Frey [33] and Aroiee and Omidbaigi [34]. They showed that N fertilization (150 kg N ha^−1^) improved leaf number, which was in agreement with Boroujerdnia and Alemzadeh [35] in their study of *Lactuca sativa* L. Similarly, Tei et al. [36] stated that the dry matter of lettuce increased with increasing N levels. Nitrogen may affect plant growth through cell division, cell enlargement, and photosynthesis, which consequently increases stem height and diameter [37,38]. The impact of nitrogen on stem height, diameter, and fresh weight was recorded by Almodares et al. [39]. Nitrogen led to increased stem length irrespective of the potassium fertilizer level. The impact of potassium was significant only when the nitrogen level was more than that of the control. As a result, the combination of N and K fertilizers could have the maximum impact on the studied characteristics. 

In the current study, the increase in shoot length, dry weight, and leaf area of cowpea lants was recorded after treatment with 50% potassium nitrate plus 50% urea, whereas the lowest dry weight was recorded after treatment with 50% N. In support, the maximum mean height of cauliflower plants was recorded in plants treated with 125% N (10.16 cm), whereas the lowest (8.88 cm) was observed with a 50% N treatment. The maximum leaf area/plant (2753.9 cm^2^) was obtained with N treatment (125% N), while the lowest (2201.3 cm^2^) was recorded with 50% N [40]. On the other hand, Shirazi et al. [41] stated that the highest maize plant height (269.40 cm) was recorded with 70 kg N/ha, while no significant differences were recorded between the control and N (120 kg N/ha) treatments. The application of 150 kg N/fed gave the maximum dry weight of the vegetative parts and a larger leaf area for broccoli [42]. Additionally, El-Masry et al. [43] reported that nitrogen fertilizers significantly increased plant growth such as leaf number, stem height, leaf area, and plant dry weight. It can be concluded that an adequate amount of nitrogen (nitrate and urea), after being applied in the soil, was taken up in the root tissues as such and, consequently, was translocated and assimilated within cowpea and sunflower seedlings, causing the observed responses (either decrease or increase in the determined growth parameters). This result is in agreement with Haroun [44]. 

### 3.2. Changes in Nitrogen Content

Our findings reveled that the trend of variation in protein content was similar to that of N content because the protein content was calculated by multiplying the N content. The protein content of seeds was progressively increased with increasing N levels [45]. In addition, the increase in protein content with increasing N levels has also been recorded in many other studies [17,46]. The results of Chandel et al. [47] proposed that the increased availability of N in rhizospheres led to an increased N uptake in rice plants. Additionally, the total grain protein content was increased under a higher N level (120 kg/h) in most rice genotypes. Soluble proteins and favorable growth conditions are improved with N supply [48]. Further, Greef [49] stated that high levels of the reduced N fraction (protein fraction) were observed in photosynthetic leaf tissue, especially under favorable nitrate supply conditions. This result proposes that the high N level increases the synthesis of amino acids in plant leaves, which has an important role on protein biosynthesis [50,51]. 

The differences in various nitrogen contents, as well as the dry weight content in cowpea and sunflower shoots and roots in response to N fertilization, are in harmony with the research of Fageria [52] and Shinano et al. [53], who stated that the accumulation of N accompanies the increase in dry matter and yield. Moreover, the application of nitrogen led to an increase in N concentrations and vegetative biomass in tomato plants [54]. From the above-mentioned pattern of changes in nitrogen contents, as well as protein contents, in cowpea and sunflower shoots and roots, in this investigation, in response to the N fertilization, there is evidence that nitrates and urea are absorbed and drawn into the metabolism of the treated plants. Thus, the absorbed nitrogen might have been, in part, responsible for transforming to amino acids and, in part, hydrolyzing to ammonia, which then combined with organic acids by amination or transamination, leading to the corresponding amino acids and, hence, being incorporated into proteins. 

### 3.3. Changes in Leaf Ultrastructure

In the current study, the decrease in the cell size of cowpea and sunflower leaves by the most used N treatments are in harmony with the research of Kano et al. [55]. Further, in the current investigation, the observed stimulation in the total size of chloroplast in the leaves of cowpea and sunflower plants in response to the different N treatments coincides with the research of Kutik et al. [56], who stated that a low N rate can cause negative changes in the ultrastructure of chloroplasts as nitrogen is a significant element of both the photosynthesis process and chlorophyll concentration. In agreement with the results of the current study, the low level of N in leaves created small chloroplasts, and Hak et al. [57] stated that high levels of N produced large chloroplasts with well-developed grana. For normal chloroplast development [56] and synthesis of thylakoid and photosynthetic enzymes, 75% of the total N in a plant is required [58]. A strong correlation has been found between the N deficiency-induced reduction in photosynthesis and the decline in ultrastructural order [59]. Moreover, the accumulation of starch granules was accompanied by a deformation of thylakoids and grana in N-deficient bean leaves [60]. The absence of starch grains in the leaves of cowpea plants, in response to those not treated with nitrogen in this study, is in accordance with the research of Bondada and Syvertsen [61], who reported that no starch granules in N-deficient leaves were recorded. They also stated that there were major changes in leaf ultrastructure, such as chloroplasts and gas exchange activities, and thus chloroplasts were small, with a low chlorophyll concentration. From another point of view, the absence of starch grains in the leaves of sunflowers, in response to nitrogen treatment with 100% urea, is in agreement with the research of Ariovich and Cresswell [62], who suggested that N can increase the mobilization of starch out of the chloroplast to sites of high carbon sink activity under higher N supply, whereas starch can build up in chloroplasts in N-deficient leaves. In the present investigation, the represented data of ultrastructure examination are supported by Lee et al. [63], who conducted an experiment to establish effective remote sensing models for plant N assessment by using different levels of N, and they examined the changes in leaf anatomical structure and chlorophyll concentration at the panicle initiation of rice (*Oryza sativa* L. cv. Tainung67). The results demonstrated that a higher N content led to a higher chlorophyll concentration and more turgid leaves. Additionally, the application of N led to improved grain yields of Yasmin rice cultivars [64] and higher yield components in durum wheat under arid regions [65]

The ultrastructure data of cowpea and sunflower plants confirmed that 50% potassium nitrate plus 50% urea treatment, in general, caused the best effect, as it recorded the highest values in chloroplast and mitochondria (number and size) that reflected positively on the various metabolic activities of the two tested plants. This is supported by the detection of the highest number of starch grains in plants that received this treatment.

Finally, a plant’s need for N is reflected in the nitrogen fertilizers, where nitrogen is accumulated in the plant in two forms—ammonia or nitrates—and influences crop species and soil conditions. This is established in this study, as the response of cowpea plants to N fertilization exceeds that of sunflower plants, which may be due to the fact that N is demanded in large amounts for sunflower growth [66]. Ammonium proved to be a source of a nitrate nitrogen equivalent when supplied at reasonable levels and with the appropriate concentration of pH buffer and a suitable level of micro- and macronutrients [67,68].

## 4. Materials and Methods

### 4.1. Plants Used

Pure strains of *V. sinensis* L. Doki cultivars (cowpea) and *H*. *annuus* L. Giza102 cultivars (sunflower) were obtained from the Agricultural Research Center, Ministry of Agriculture, Giza, Egypt. All chemicals used in this investigation were of analytical grade. The used potassium nitrate and urea fertilizers were obtained from the Ministry of Agriculture, Giza, Egypt.

### 4.2. Time Course of Experiment

A homogenously volumed lot of cowpea and sunflower plants were selected and surface sterilized by soaking them in a 0.01% HgCl_2_ solution for 3 min. After washing thoroughly with distilled water, the seeds of each plant were divided into 5 equal groups, each containing 100 seeds, and treating them (by irrigation) as follows:

Group (1): left to grow without fertilization to serve as a control.

Group (2): fertilized twice at 10 and 30 days from sowing with potassium nitrate (in concentrations of 100% of the recommended dose; 1 g/pot).

Group (3): fertilized twice at 10 and 30 days from sowing with urea (100% of the recommended dose; 0.6 g/pot).

Group (4): fertilized twice at 10 and 30 days from sowing with potassium nitrate and urea (both in concentrations of 50% of the recommended dose; 0.5 g nitrate plus 0.3 g urea/pot). 

Group (5): fertilized firstly at 10 days from sowing with potassium nitrate (in a concentration of 100% of the recommended dose; 1 g/pot) and secondly at 30 days from sowing with urea (in a concentration of 100% of the recommended dose; 0.6 g/pot).

The experiment was approved in the greenhouse of the Botany Department of the Faculty of Science, Mansoura University. Ten seeds of the five groups were cultivated in pots (30 cm in diameter) with equal amounts of soil (sand: clay, 1: 2 *v*/*v*). The physical and chemical analyses were carried out on the used soil samples, with results as follows: permanent wilting point (%): 17.00; available water (%): 10.00; clay (%): 26.89; sand (%): 50.35; silt (%): 22.76; total carbon (%): 3.33; organic matter (%): 5.75; calcium carbonate (%): 0.45; pH: 8.21; electrical conductivity (dsm^−1^): 2.046; and soil texture class: clay. The pots were irrigated using the usual practice by adding equal amounts of water to each pot, when required. All plants were exposed to normal day and night conditions (13 h light and 11 h dark at 32 °C ± 2 and 20 °C ± 2, respectively, with 58% relative humidity). After 15 days, thinning took place where only five uniform seedlings were left to grow in each pot. The objective of this experiment was to evaluate the effect of different nitrogen sources and levels on leguminous and non-leguminous plants, and so the cowpea and sunflower plants were selected according to their economic importance. Further, the stage of this study was the vegetative stage, and thus the samples were taken at 50 days from sowing, before flowering stage, with three replicas for each, but the mean is tabulated. These samples were used for assessment of the shoot growth parameters, relative growth rate, net assimilation rate, and the nitrogenous constituents of shoots and roots. In addition, transmission electron microscopy was carried out for only one sample of leaves.

### 4.3. Analytical Studies

The relative growth rate of the shoots was determined according to Jose and Gillespie [69] over a period of known days by the formula:

Relative growth rate of shoot = Ln (final dry weight minus initial dry weight)/period in days.

The net assimilation rate is calculated as the increase in plant dry weight per increase in leaf area and unit time [70], as follows:

Net assimilation rate = (final dry weight minus initial dry weight)/(final leaf area minus initial leaf area)/period in days.

Estimation of nitrogenous constituents

The extraction method was adopted from Yemm and Willis [71] as follows:

The dried samples were ground to powder, then a known weight was extracted in distilled water by grinding the samples at room temperature for 30 min in a glass mortar. The mixture was then transferred to a boiling tube and brought quickly to a water bath for 15 min at 80 °C. The insoluble residue was removed by filtration, then the filtrate was made up to volume and used for the estimation of the different nitrogen fractions.

Determination of total N

The total N was determined as ammonia by the conventional semi-micropropagation of the Kjeldahl method of Rees and Williams [72] and described by Haroun [73]. Ammonia-N was assessed spectrophotometrically using Nessler’s reagent by the method adopted by Delory [74] and modified by Naguib [75] as follows:

Exactly 0.02 to 0.03 g of the dry powdered tissue was heated for at least 8 h with 0.5 g catalyst (K_2_SO_4_: 80 g; CuSO_4_ 5H_2_O: 20 g; and SeO_2_: 0.3 g), 2 mL of ammonia-free concentrated H_2_SO_4_, and 1 mL of distilled water. The solution was treated with 15 mL of 40% NaOH and steam-distilled in the conventional manner into 5 mL of 0.05 N H_2_SO_4_. The distillate was made up to volume and used for the estimation of the total nitrogen by estimating ammonia.

Determination of total soluble N

The total soluble nitrogen was determined by the Kjeldahl method in Pirie [76] and described by Haroun [77] as follows:

A known volume of the extract was taken into a digestion flask and heated for at least 8 h with 0.5 g catalyst (K_2_SO_4_: 80 g; CuSO_4_.5H_2_O: 20 g; and SeO_2_: 0.3 g), 2 mL of ammonia-free concentrated H2SO4, and 1 mL of distilled water. The solution was treated with 15 mL of 40% NaOH and steam-distilled in the conventional manner into 5 mL of 0.05 N H_2_SO_4_. The distillate was completed to a known volume using distilled water and the total soluble nitrogen was estimated as ammonia, as described earlier.

Determination of insoluble protein nitrogen

The insoluble protein nitrogen was calculated as the subtraction of the amount of total soluble nitrogen from the amount of total nitrogen of the same sample, according to A.O.A.C. [78]. 

### 4.4. Transmission Electron Microscopy (TEM)

#### 4.4.1. Fixation

Tiny sections (4 mm^2^ × 4 mm^2^) from the mature leaves of *V. sinensis* (Cowpea) and *H. annuus* (Sunflower) plants at only the second vegetative stage were used for electron microscopy. The specimens were fixed in the primary fixative 4F1G (1% glutaraldehyde and 4% formaldehyde in 0.1 M phosphate buffer PB, PH 7.4) for at least 120 min at room temperature, followed by washing in 0.1 M PB three times for 15 min each time. Then, the fixation was completed in osmium tetroxide for 60 min in 0.1 M PB.

#### 4.4.2. Dehydration

Dehydration was completed via a series of 30%, 50%, 70%, and 95% ethanol and, finally, absolute ethanol for 15 min immersion in each concentration. The ethanol was replaced with acetone via rinsing in acetone two times for 15 min for each time. 

#### 4.4.3. Infiltration

The dehydrated samples were infiltrated with Embed 812 via a stepwise series of Embed 812: acetone (1: 1) for 1–2 h, followed by rinsing in Embed 812: acetone (2: 1) for overnight in a desiccator. Finally, the samples were polymerized in pure Embed 812 resin overnight at room temperature and then heated for 24 h at 60 °C in an oven. 

#### 4.4.4. Sectioning

Mounted blocks were trimmed with razor blades. After trimming, glass knives were used to make ultrathin sections using an ultramicrotome (RMC_ power tome XL/USA), which were then collected onto grids. Plastic tape was used, and the tape was sealed to the glass with dental wax.

Semi-thin sections (500 nm thickness) were cut using glass knives and floated on water surfaces, then picked up and placed on a drop of water on a slide. The slide was heated and the sections adhered to the slide. A drop of toluidine blue stain was placed on the section for 90 s. Then, they were washed with distilled water and the sections were examined by light microscope. Ultra-thin sections (100 nm thick) were cut using glass knives and the sections were floated on water surfaces in the form of a ribbon. Silver-gray or gold high quality sections were cut. The sections were manipulated to the center of the boat prior to being picked up on grids.

#### 4.4.5. Grid Preparation

Copper hexagonal mesh with 2.05 mm grids were degreased by washing them in chloroform, and they were stored on filter paper in a glass petri dish.

#### 4.4.6. Staining

Double staining was completed in uranyl acetate followed by lead citrate.

Uranyl acetic acid derivation stain

An immersed fluid arrangement of 5% uranyl acetic acid derivation (nearly 5 g/100 mL H_2_O) was used as the stain. The stain was shaken for a time, then 10 drops of glacial acetic acid were added. After that, the solution was stored in brown glass away from light. Staining with uranyl acetate was carried out by putting drops of the stain on a square of dental wax, and then each grid was floated onto a drop of the stain with the section facing the stain. The sections on the grids were stained for 15 min in the dark and then washed with distilled water and left on filter paper to dry.

#### 4.4.7. Reynolds Lead Citrate Stains

Lead nitrate (1.33 g) and sodium citrate (1.76 g) were mixed with 30 mL distilled water and shaken for 1 min, followed by shaking for 30 min. To this solution, 8 mL of a 1 N solution of sodium hydroxide was added and mixed by inversion until the solution became clear. Distilled water was added up to a final volume of 50 mL. The sections were stained with lead citrate in a carbon dioxide-free atmosphere. A square of dental wax was placed onto a petri dish containing filter paper and 3–4 pellets of sodium hydroxide were added to prevent the formation of lead carbonate and to absorb carbon dioxide. The lead citrate solution was centrifuged for 15 min at 5000 rpm. A few drops of the supernatant were transferred onto the wax. A single grid was floated onto each drop of the stain for 15 min. The sections were washed with 0.02 N sodium hydroxide and distilled water, then left on a filter paper [79].

#### 4.4.8. Examination with TEM 

The stained sections were examined using a JEM—JEOL 2100/Japan transmission electron microscope.

#### 4.4.9. Cell Structure Analysis

The public domain Image J software package was used for analyzing the size of cells and other organelles http://rsb.info.nih.gov/ij/ (13 February 2022)

The obtained images of examination on the TEM were applied on the http://rsb.info.nih.gov/ij/ (accessed on 13 February 2022) software package that was used for analyzing the size of cells and other organelles.

### 4.5. Statistical Analysis

The data were analyzed by the least significant difference (L.S.D) test at a probability of 0.05 to identify the significant effect of a treatment. ANOVA analysis was completed with the IBM SPSS-20 statics software [80].

## 5. Conclusions

In conclusion, our results confirmed that nitrogen fertilizers have an overall positive correlation with the determined physiological aspects and ultrastructure of the tested plants, especially in the case of using 50% potassium nitrate plus 50% urea, and the magnitude of response was more pronounced in the case of the cowpea plant as a representative leguminous plant, as sunflowers demanded larger amounts of N. Thus, on the basis of this fact that, up to now, there has been a demand for reducing the environmental pollution that results from the over-application of nitrogen (N) fertilizers. This investigation suggests that using N fertilization in a concentration of 50% potassium nitrate plus 50% urea provided the tested plants with sufficient nitrogen content for normal, healthy growth, which was confirmed by the best results of shoot length, dry weight, shoot water content, relative growth rate, and net assimilation rate in cowpea plants. In addition, N fertilization in a concentration of 50% potassium nitrate plus 50% urea gave the best results of the most studied characteristics of sunflower plants, especially shoot length, relative growth rate, net assimilation rate, number of nodes/plants, and shoot dry weight. Additionally, the leaf ultrastructure of cowpea and sunflower plants were improved with 50% potassium nitrate plus 50% urea compared with the control and other treatments. This experiment was carried out in pots that are somewhat similar to what happens under field conditions. 

## Figures and Tables

**Table 1:** Effect of the two levels of potassium nitrate and/or urea on the growth parameters of *V. sinensis* and *H. annuus* at the vegetative stage (after 50 days from sowing).

Plant	Treatments	Parameters								
		Shoot Length (cm)	Number of Nodes/Plants	Number of Leaves/Plants	Leaves Area/Plant (cm^2^)	Shoot Fresh Weight (g)	Shoot Dry Weight (g)	Shoot Water Content	Relative Growth Rate	Net Assimilation Rate
*V. sinensis*	T1	19.375	5.375	10.75	174.878	5.121	0.753	4.368	0.062	0.00015
	T2	22.675 *	6	12	161.264	6.12	0.888	5.232	0.065	0.0002
	T3	21.75	4.875	9.75	144.43	6.494 *	0.895	5.599 *	0.078	0.00023 *
	T4	22.063 *	4.875	9.75	203.872	7.788 *	1.248 *	6.540 *	0.084 *	0.00025 *
	T5	21.788	5.25	10.5	235.402 *	6.486 *	0.895	5.591 *	0.065	0.00014
*H. annuus*	T1	47.563	4.875	9.75	700.471	4.009	0.556	3.453	0.055	2.438
	T2	53.625	8.375 *	16.750 *	1560.98 *	9.639 *	1.198 *	8.441 *	0.063	2.498
	T3	55.500 *	9.500 *	19.000 *	1796.246 *	12.707 *	1.549 *	11.158 *	0.077 *	3.017
	T4	57.620 *	9.000 *	18.000 *	742.475	11.769 *	1.606 *	10.163 *	0.078 *	7.995 *
	T5	50.188	8.750 *	17.250 *	1723.659 *	8.429 *	1.090 *	7.339 *	0.071 *	2.163

(*) = significant increase or decrease at 0.05 LSD; T1: control; T2: 100% potassium nitrate; T3: 100% urea; T4: 50% potassium nitrate plus 50% urea; T5: 100% potassium nitrate plus 100% urea.

**Table 2:** Effect of the two levels of potassium nitrate and/or urea on the nitrogen fractions (g/100 g dry weight) of *V. sinensis* and *H. annuus* shoot at the vegetative stage (after 50 days from sowing).

Plant	Treatments	Parameters		
		Total Nitrogen	Insoluble Protein Nitrogen	Total Soluble Nitrogen
*V. sinensis*	T1	14.538	9.813	4.725
	T2	9.026 *	6.093 *	2.933 *
	T3	9.495 *	6.409 *	3.086 *
	T4	14.875	10.041	4.834
	T5	6.932 *	4.679 *	2.253 *
*H. annuus*	T1	6.986	4.716	2.270
	T2	13.359 *	9.017 *	4.342 *
	T3	12.276 *	8.286 *	3.990 *
	T4	7.094	4.789	2.305
	T5	14.598 *	9.854 *	4.744 *

(*) = significant increase or decrease at 0.05 LSD; T1: control; T2: 100% potassium nitrate; T3: 100% urea; T4: 50% potassium nitrate plus 50% urea; T5: 100% potassium nitrate plus 100% urea.

**Table 3:** Effect of the two levels of potassium nitrate and/or urea on the nitrogen fractions (g/100 g dry weight) of *V. sinensis* and *H. annuus* root at the vegetative stage (after 50 days from sowing).

Plant	Treatments	Parameters		
		Total Nitrogen	Insoluble Protein Nitrogen	Total Soluble Nitrogen
*V. sinensis*	T1	0.880	0.594	0. 286
	T2	0.950 *	0.641 *	0.309 *
	T3	1.020 *	0.688 *	0.332 *
	T4	1.240 *	0.837 *	0.403 *
	T5	1.050 *	0.708 *	0.342 *
*H. annuus*	T1	0.940	0.634	0.306
	T2	0.350 *	0.236 *	0.114 *
	T3	2.380 *	1.606 *	0.774 *
	T4	1.401 *	0.946 *	0.455 *
	T5	1.960 *	1.323 *	0.637 *

(*) = significant increase or decrease at 0.05 LSD; T1: control; T2: 100% potassium nitrate; T3: 100% urea; T4: 50% potassium nitrate plus 50% urea; T5: 100% potassium nitrate plus 100% urea.

**Table 4:** Means of the cellular and sub-cellular measurements of *V. sinensis* leaves at the vegetative stage (after 50 days from sowing) in response to the two levels of potassium nitrate and/or urea.

Treatments	Parameters							
	Cell Size (µm)		Cell Wall Thickness (µm)	Cytoplasm Size (µm)		Vacuole Size (µm)	Nucleus Size (µm)	
T1	165.25		0.0022	80.145		85.112	13.827	
T2	139.15		0.0081	60.537		78.617	25.910 *	
T3	325.86		0.0097	204.031 *		121.835	16.582	
T4	573.13 *		0.012 *	210.661 *		362.442 *	17.296	
T5	144.38		0.004	60.96		83.424	6.407	
**Treatments**	**Chloroplast**			**Starch/Chloroplast**		**Mitochondria**		
	**NO**	**Size** **(µm)**	**Total Size** **(µm)**	**NO**	**Size** **(µm)**	**NO**	**Size** **(µm)**	**Total Size (µm)**
T1	6	4.787	28.722	2	0.753	4	0.796	3.184
T2	9.0 *	7.967 *	71.703 *	2	1.869 *	8 *	0.834	6.672 *
T3	0.10 *	8.009 *	80.09 *	0	0	3 *	0.551	1.653
T4	0.10 *	14.058 *	140.058 *	3 *	5.220 *	5 *	0.508	2.54

(*) = significant increase or decrease at 0.05 LSD; T1: control; T2: 100% potassium nitrate; T3: 100% urea; T4: 50% potassium nitrate plus 50% urea; T5: 100% potassium nitrate plus 100% urea.

**Table 5:** Means of the cellular and sub-cellular measurements of *H. annuus* leaves at the vegetative stage (after 50 days from sowing) in response to the two levels of potassium nitrate and/or urea.

Treatments	Parameters							
	Cell Size (µm)	Cell wall thickness (µm)	Cytoplasm Size (µm)	Vacuole Size (µm)	Nucleus Size (µm)			
**T1**	146.901	0.005	85.13	61.771	11.541			
**T2**	260.098 *	0.007	177.676 *	82.421	11.976			
**T3**	110.533	0.006	41.737	68.796	10.987			
**T4**	57.984	0.005	34.674	23.81	10.446			
**T5**	220.996	0.017	107.966	113.030 *	14.924			
**Treatments**	**Chloroplast**			**Starch/Chloroplast**		**Mitochondria**		
	**Number**	**Size (µm)**	**Total Size (µm)**	**Number**	**Size (µm)**	**Number**	**Size (µm)**	**Total** **Size (µm)**
**T1**	8	5.548	44.384	0	0.000	4	0.216	0.864
**T2**	13.000 *	4.216	54.808	1	0.424	3 *	0.430	1.290
**T3**	8	4.066	32.048	1	0.286	4	0.456 *	1.824 *
**T4**	14.000 *	3.331	46.634	2	0.244	6 *	0.384	2.304 *
**T5**	6.000 *	8.825*	52.950	1	0.268	3*	0.450 *	1.350

(*) = significant increase or decrease at 0.05 LSD; T1: control; T2: 100% potassium nitrate; T3: 100% urea; T4: 50% potassium nitrate plus 50% urea; T5: 100% potassium nitrate plus 100% urea.

## Data Availability

Not applicable.

## Abbreviations

N	nitrogen
K	potassium
N/ha	nitrogen/hectare
kg N/fed	kilogram nitrogen/feddan
kg/h	kilogram/hectare
hrs	hours
min	minutes
mm	millimeter
g	gram
